# Foreign Medico-Psychological Literature

**Published:** 1861-10

**Authors:** 


### FOREIGN MEDICO-PSYCHOLOGICAL LITERATURE.

OUR Retrospect of current Medico-Psychological Literature will embrace the following subjects:

1. On the Moral Responsibility of Lunatics.
2. On the Statistics of Insanity.
3. On a case of Epileptiform Convulsion.

1.—*On the Moral Responsibility of Lunatics.*—By DR. H. BELLOC, Superintendent Physician of the Department Asylum of Alençon (Omer).

IN the April number of this Journal we recorded certain conclusions of Dr. Belloc upon this subject. In the last number of the *Annales Médico-Psychologiques*, Dr. Belloc, *apropos* of a medico-legal report on a case of parricide, discusses the bearing of his opinions upon the duties of the alienist physician in the witness-box. He remarks first on the unfortunate antagonism which too commonly arises, in questions of lunacy, between the representative of medicine and the representative of the law. The source of this antagonism Dr. Belloc seeks in the different significations which the medical man and the lawyer persistently attach to the terms used in law to designate lunacy, and to the false position which, as a consequence, is so frequently forced upon the former by the requisites of special pleading. The one refuses to recognize lunacy except in its medical sense; the other except in its legal. Dr. Belloc then proceeds:—" If it were a question of seeking scientifically and theoretically the distinctive character of mental alienation without regard to any determinate application, the physician would be right. He would then only be concerned with the nature of the malady without reference to its extent, and would be obliged to pronounce the man mad who is under the dominion of a single delirious fancy, as much as another who has reached the last stage of distraction. But he does not do so. In fact the question of extent becomes of supreme importance and decides the whole question.

It, alone, gives to an act its true character, and is the measure of its morality. It is for want of making this fundamental distinction that physicians, in my opinion, have been beside the question. The minister of public affairs does not ask us whether the accused person, whom he presents to us, would be declared mad by the testimony of a *concours* or an academy! To examine things thoroughly, he does not first occupy himself with that; but with a very different assertion that the culprit, in committing the act of which he was accused, 'knew what he was doing, and might have prevented himself from doing it.' And we doctors, of this simple practical question make a nosological question, and we think we have answered it when we say, the accused person is under delusion on this point, therefore he is mad.

"That which astonishes me is, that the magistrates have not till now perceived the logical error involved in this *passage d'un genre à l'autre*, which we have for so many years invariably committed. They have not seen that we answer that which they do not ask of us, and that we do not answer that which they do ask. Hence the indefinite prolongation of *quid pro quo;* hence the interminable antagonism between the opinion of the bench and the opinion of the physician; an antagonism which puts to the rout good faith and good sense, and which scandalizes the populace, which is carried so far that nothing is seen but the strife of self-conceit and party-spirit in that which is in reality only the necessary consequence of a misunderstanding.

"The magistrate and the physician are here, each on his own side, victims of an educational prejudice, which causes them both to hesitate in saying all that they think. In a great number of cases the first sees clearly that the accused advances certain eccentric, extravagant, and, at least, singular ideas; but, in his sympathy with popular indignation, he refuses to recognise in these the real signs of madness; for, he thinks, if I allow that the accused raves on a single point, it will be decided, *ipso facto*, that he is not guilty, and I believe in my soul and conscience he is guilty! The physician, on his side, sees clearly that the accused has considered his act; that he has discussed it with sagacity; that he has weighed its consequences; that he has calculated the necessary means; that he has decided on it, and perpetrated it, with absolute determination to do it. But, he says to himself, if I allow that, he will be judged as if he was not mad; and in my soul and conscience I believe him to be mad!

"We may see how, on this ground, or, we may more justly say, on these different grounds, the two adversaries can never meet each other.

"How then can we bring them face to face in a way that the arguments will justify, in order to bring truth to light, and to ensure the triumph of justice, to which all other interests should be sacrificed?

"Nothing, in my opinion, is more simple. It is only necessary to abandon the fictions that have so long taken the place of realities—the absolute definitions that have made a malady according to programme, a malady all of a piece, of a series of pathological alterations essentially diverse, essentially variable, and essentially varied in their intensity, in the mode and in the number of their manifestations, as they are varied in their causes. It is sufficient to declare frankly and loudly that nature does not recognise the rectilinear limitations in which the feebleness of our intelligence takes refuge; that she does not regulate her acts to accommodate our classifications; that nothing is more rare than absolute dementia in a legal sense; that the greater number of lunatics, although really lunatics, retain for many of their actions the whole, or a part, of their free-will; that physicians can say only that which is the result of their own observations, and that, consequently, it cannot be reasonable to question whether such and such accused person is absolutely wise or absolutely senseless, absolutely responsible or absolutely irresponsible. But *what are in his case the limits within which society may, without injustice, expect him to account for his actions?* Then magistrates and medical men will be able to discuss the question with the hope of understanding it; then will become more and more rare these arguments without issue, these condemnations without mercy, and these unhappy acquittals which, too often, cause the true friends of humanity to tremble.

"Let not my brethren condemn too hastily my opinion, though it may astonish many of them. I believe a little reflection will render it less paradoxical in their eyes.

"There is nothing new in what I have just said, except the application I have made of a truth well known to every physician who has lived in an asylum. What would become of us who have the management of the insane if the doctrine of absolute irresponsibility should prevail for an hour? Is not all our influence, all our conduct based on the capacity of the insane to understand the counsels given to him, and the reprimands addressed to him, and to control himself accordingly? Every day in the asylum of which I have the management I praise, I reward, I blame, I constrain, I threaten, I punish; and why? am I then myself an idiot?

"And that which I do all my colleagues do also, and from the very necessity of the case; and what does this prove but that the threat of a punishment, that the assurance of a real responsibility may combat, perhaps effectually, in the minds of certain lunatics the first delirious idea, the first thought of a crime, before it has entirely mastered the powers of resistance. And that if certain lunatics are not so guilty as the magistrate supposes, no more are they altogether innocent, as is asserted by the physician.

"In the face of these incontestable facts, in the face of our daily practice, what becomes, I ask, of the doctrine of absolute irresponsibility on which we insist in a court of justice? In truth it is not believed in, and I can only explain this flagrant contradiction by the spectre of the guillotine which the minister of public affairs continues to hold up before our eyes. In the presence of this supreme danger, which is incurred by one class of our invalids—the class in whose safety we feel ourselves most interested, it has appeared to us that we could not do too much to avert it, and in the fear of failing to accomplish our end we have, without perceiving it, passed beyond the limits of reason and justice. Fearing that the hesitation of our mind might damage our cause, we have endeavoured to conceal it, and not being able to fix with absolute certainty the responsibility incurred, we have declared that there was no responsibility at all. This is, I think, the secret, and the excuse of the logical mistake into which we have fallen.

"But we cannot persist indefinitely in this sophism, and it is time, and it is only strict justice to recognise in law that which we have for so many years recognised in fact.

"Besides all this, to avoid self-deception, I request once more the attention of my colleagues. Besides the question of equity, I find here involved a question of philanthropy. The doctrine of irresponsibility which seems at first sight so favourable to lunatics, has in reality done them more harm than all judicial errors put together. Is it not this doctrine which has originated the *régime* of grated cells and chains of iron?—the only mode of management that can be logically employed against those individuals who are regarded as absolutely incapable of self-control—as nothing more than brute beasts. And, in relation to the number and in relation to the magnitude of the evils endured, what are the victims of judicial errors compared with the victims of chains and dungeons? Two men, whom I associate in the same honour, as I do not know whether one was anterior to the other, Pinel and Daquin, have attempted the task of putting down this frightful system; and what has been their *point de départ?* They commence by affirming, after long and attentive investigation, that the greater number of the insane are not, as was formerly supposed, mere machines; that they are accessible to reasoning, to good sentiments; that they can, in the greater number of cases, distinguish the true from the false, the just from the unjust; that they possess in consequence a certain amount of moral freedom. This being the case, reform becomes practicable, otherwise it is an absurd impossibility.

"Unhappily this starting-point has escaped the notice of the successors of Pinel; and I venture to say, notwithstanding the respect I have for them, that it is for want of returning to the inspiring thought of their master, that stopping half-way on the road that he has indicated, they have allowed themselves to be again entangled in the doctrine of absolute irresponsibility, while they strive to resist its consequences.

"This error incontr avening their benevolent designs has embarrassed the manifestation of these designs, and has prevented them, in spite of all their efforts, from rising to any higher conception than that of an actual asylum. Heaven forbid that I should deny the services that have been rendered by these establishments. I am glad to proclaim my affection for them and my admiration for their founders; but while I heartily acknowledge the amelioration that they have introduced into the *régime* adopted in former days, I cannot avoid the conviction that they perpetuate its traditions; what is in fact the system of barrack accommodation that they adopt but a sort of prolongation of the chains of former times? Nothing can prevail against this fundamental defect—neither the benevolent intentions of ministers and magistrates, nor the confiding generosity of general councils, nor the devoted attention of medical men. All principles must necessarily bring sooner or later the consequences by which they will be judged; and the system under consideration, after having procured to the insane a little more air, a little more light, a little more relative liberty, more regular meals, more frequent change of linen, and other improvements of detail, what has it produced after all? It has produced that which it might be expected to produce, that which was logically, fatally contained in its systematic divisions, in its paved courts, in its regular colonnades, in its enclosures of high walls, the material expression of its starting-point; it has produced an administration altogether mechanical, with which I will not concern myself at present. It is sufficient to say, in order to establish that which I have advanced, that in extinguishing all variety, in stifling every germ of innovation, it supposes that the last word is said upon the arrangement and the management of asylums; that by it they are rendered immoveable, and condemn generations on generations of insane to perpetual incarceration. Can such an embargo upon the development of the views of Pinel and Daquin be intended by the supporters of these two friends of the insane?

"Let not, then, an ill-understood philanthropy arrest our progress. Let us fearlessly remove the evil spread before our eyes which hides from us the truths evident to simple common sense. In doing this, far from injuring those whose cause we advocate, we cannot fail to be useful to them. In declaring that the lunatic can and ought in certain cases to bear justly a part of the responsibility, I am well aware that we partially condemn the partially guilty who on theory would be declared innocent; but at least in turning our back on the past, we shall have opened the door not only to simple improvements but to a true reform—a reform real enough this time—a reform indefinitely progressive, for it has its principles in nature itself, and wants only time for its development. We shall, in fine, have prepared in the future for the greater number of insane a *régime* of dignity, of liberty, of domestic life, of family affection, of well-being, and of physical and moral development, of which the theory of irresponsibility and its necessary mode of application deprives them now even of the hope."

---

### 2.—*On the Statistics of Insanity.*

A writer in the *American Journal of Insanity* (July, 1861), offers the following suggestive remarks on this subject:—

"It has not been sufficiently considered, we think, that the subject of insanity has two widely different aspects, whence it is to be studied from separate, and in some respects opposite, points of view. These are the medical, and the social or political. The practical and administrative parts of these two divisions cannot, and need not, always be separated, but their scientific relations are entirely dissimilar, and, we are convinced, can only be studied apart from each other with any prospect of success. This will be evident from a brief view of the subject of statistical records respecting the insane. We have already referred to the origin of statistical science, and need only allude to the rank which it has attained in the forms of abstract as well as of applied knowledge. It has survived the attacks of ridicule and abuse, until it has come to be acknowledged as the only safe basis of social and governmental reform, of commercial enterprise, and indeed of all the grandest schemes of human progress. Books of statistics, instead of being what Lamb once wittily, and with something of truth, termed them, 'books that are not books,' are now much more truly those from which, or because of which, books are made. But statistical science is not, as seems too often supposed, a mere record of heterogeneous facts. This were indeed 'learning made easy,' to the meanest intellect. The facts of statistics must, in the first place, be such as are capable of being reduced to numerical expressions with a degree of exactness. These, of course, are only of the most general and collective classes. In proportion as facts become less general is their statistical value reduced, until we reach the individual and special classes, which are worthless. Again, facts should be collected and arranged with a knowledge of the purposes to which they are to be applied. A consideration with which idle collectors of empty facts often flatter themselves is, that the statist must not deal with application or speculation, which would unfit him for observing properly. True, it is not the part of the statist to combine or to apply facts, but it does belong to him to point out how they may be combined, and what principle must govern their application. The neglect of this has done much to bring the method into disrepute, and to give rise to the saying, that 'anything may be proved by statistics.'

"Let us now look at the forms of statistics usually adopted in the specialty of psychological medicine. And, as at once the most convenient and the most perfect collection of the kind recently made, we will first take the abstract of 'Statistics of the Establishments for the Insane in France, from 1842 to 1853 inclusive," published in this journal for April 1860 and 1861. A highly centralized administration, having for its head in each department the first scientific men of a country, must ensure the great advantages of a thorough system of records, and the selection of competent observers. Now the number of institutions for the insane of a country, their capacity, their mode of support, increase, distribution, population, with their admissions and discharges at a certain time, and at regular periods, are seen at once to be capable of exact numerical expression, and when compared with such other facts as the whole number of insane, and the total population, afford the most perfect guide to the legislator and the political economist. This is one kind of the facts given, and is truly a matter of state or collective concern. Another class has an indirect importance of the same character. These are such as the nativity and residence of the patients, the numbers recovered and not recovered, the number of re-attacks, and the number of deaths. They do not, like the others, point to necessary conclusions, but they afford ground for more or less probable inferences, which may be tested by other series of facts, and these by others still, according to the degree of complexity in each case. In the nativity of patients, for instance, there can be no direct practical interest. The spot of earth upon which an individual was born, can have no more relation to a state of mental disorder in his case, than the shape which the clouds will assume on the day of his death. The fact of race, which might be implied, could have little greater pertinency. Yet by comparing the number of insane of a foreign with those of native birth, and comparing the total native population with the whole number of immigrants, and in a similar way guarding against several obvious sources of fallacy, an inference might perhaps be derived, in the most general terms, as to the health and vigour of the entire foreign element from which the number of insane were derived. Still, it is plain that our statistics would be only an indirect and unimportant contribution toward such an estimate.

"The third variety of facts contained in these statistics comprises all those concerning the vocation, degree of education, civil condition, residence, sex, age, season of attack, of recoveries and of deaths, vocation of recovered and of deceased, and other similar particulars. We have omitted to include the etiological records, which will be hereafter noticed. Now let us ask ourselves for what purpose these facts have been drawn out with such an ingenious minuteness. Their collocation cannot be based upon any principle of statistical science. They can be recorded numerically, but figures carry no virtue not contained in terms; and the common principle by which they are possibly connected must be expressed, before the first step in their combination is made. For it is to be remembered that no useful observation is possible, except as connected with some known or assumed principle which is present to the mind of the observer. Bald facts, without some hypothesis to co-ordinate them, are the merest rubbish. Even in physical science, where general laws are so accurately known, and deductive conclusions do so much to guide the student, still some hypothesis must attend upon the severest inductive processes in the discovery of special laws. How much more, then, do we need theory in organic science, whose most general laws are beyond our grasp. We have in meteorology a science whose foundation principles are capable of the most exact mathematical expression, but whose data are so extremely numerous and complicated as to render its progress by pure induction quite impossible. Who does not know the large use which is made of theory in the pursuit of this study? Yet in the study of mental phenomena, whose data are infinitely more complex, and whose primary laws are unknown, we are cautioned against anything beyond the merest record of facts.

"If any proof were wanting of the extreme fallacy of the grouping of facts in connexion with insanity, like those which we are now considering, it might be found in the comments which accompany these statistics. As though with tacit assent to their meaningness, their obvious meaning is coolly reversed or negatived whenever—as is often the case—it is contrary to, or not confirmed by, the preconceived views of the writer. Thus instead of affording a test to theory, these facts are merely the excuse for offering endless hypotheses in their explanation. Let us take, for instance, the first table in the second part of the article referred to as given in our last number. This table shows that, during a given period, the yearly number of discharges from French asylums gradually diminished. Now the data given here we suppose to form a part of the true statistics of a country. Taken together with the facts that the admissions to asylums for the same period had increased nearly 300 per cent., and that the number and capacity of these institutions had increased to a definite extent, we have a reliable basis for a few general statements of certain value. But M. Legoyt evidently fears that, as is the manner of psychologists, we must infer from these data a decreased efficiency of treatment, and thence goes on to explain as follows:—

"'We regret that the documents do not furnish the means of determining exactly whether this reported diminution belongs to the patients discharged before or after recovery. Still, we do not hesitate to admit the former hypothesis, and conclude that many were discharged before their complete recovery would warrant. For, on the one hand, it is not easy to believe — especially when we take into account the new therapeutic resources and the increased comforts which belong to nearly all modern asylums—that the treatment of the insane is less effective than formerly; and, on the other hand, it is probable that families, appreciating more fully from day to day the great advantages of these institutions, are more and more disposed to maintain their friends in asylums, even after they may be acknowledged as incurable cases.'

"The table above referred to also shows what is termed 'a very interesting fact,' which is, that the percentage of discharges from asylums has been greater among males than among females. Instead of noticing this as a natural consequence of the larger yearly number of male admissions, the writer observes that 'the explanations of this excess offered by directors of asylums have been various,' and proceeds to enumerate some of them; but he himself questions 'whether we ought not rather to attribute this difference to the greater or less severity of the disease itself, depending upon the difference in causes which induce insanity in the two sexes.' Here is implied the error which has been before alluded to, of comparing things numerically which can have no numerical expression. The terms insanity, disease, and cause—when the latter is predicated of vital phenomena—are not entities which may be dealt with by numbers. But we shall have occasion to allude to this again. It is enough to say, there is no ground in reason or analogy for the primary assumption that sex has any necessary and positive relations to insanity. The presumption is, then, that it has none; and there is nothing in all the figures which have accumulated upon the point to weaken that presumption in the least. Yet the table giving the number of admissions according to sex, M. Legoyt says, 'seems to settle the question' of the relative liability to mental disease. It has been thus settled, as our readers are well aware, very many times, on one side and on the other. Will not some enterprising statistician at length draw up these opposite conclusions numerically, and strike a balance which shall be final—until the next report of asylum statistics is published? Yet it is satisfactory to see that, at points where we might expect some such absurd inference as the above, the writer is very happy in the common-sense explanations to which he limits himself, and which are, as he says, 'wholly independent of psychological influences.'

"The fallacy, or rather the folly, of this array of heterogeneous facts in the name of the numerical method, may be further illustrated by the various tabulation of the ages of patients, with the several particulars of admission, recovery, death, &c. This comparison is made because of the hypothesis that age has an influence in the development of insanity. Now what shadow of excuse is there for any such supposition? It is proper that we should be reminded here, that an empirical etiology has always gone before a scientific one, which has had only to render the former more exact and certain. Centuries ago the Father of Medicine remarked that phthisis was most commonly developed between the ages of thirteen and thirty-five years. The widest statistical records, made within the last century, have simply served to render this relation in more precise terms, and to refer to an imposing mass of figures as its proof. This may have been of some use in the construction of life tables, although to medical men it can have only an infinitesimal value. But no physician, from the time of Hippocrates down, has observed that insanity belonged to any particular time of life more than another. And this is not to be wondered at, when the vaguely defined condition which we call insanity is contrasted with phthisical disease. Death and certain organic lesions are the test in one case. What it is in the other, who can yet tell us?

"We may mention here a curious instance of the empirical suggestion of causes in insanity. Probably most of our readers have looked over the numerical inquiries once made into the supposed influence of the moon in causing mental disorder, and in varying its symptoms. The observation of numerous and intelligent persons upon this point for centuries was enough to prompt the most searching and methodical inquiry. The statistics, if we recollect aright, indicated some sort of connexion between the moon's phases and morbid mental symptoms much more strongly than have those of the various accidents of age, sex, vocation, &c., with the same. But these coincidences were explained away, to the satisfaction of nearly all except the most ignorant and superstitious. This came, of course, in the progress of astronomy. In spite of statistics, and, indeed, of superficial observation to this day, the theory of lunar influence has ceased to receive attention. Have we not advanced far enough in meteorology, if not to create a disbelief in the effect of climatic changes upon insanity, at least to convince us of the utter hopelessness of further inquiry? Add to the immensely numerous and complicated data of this science the intangibilities of mind in its most perplexing manifestations, and do not our records which give the admissions, deaths, &c., according to season and month, appear fanciful to the last degree?

"To other statistical heads of the same character as the above we will only refer. The tables of civil condition, vocation, and education are the principal ones not already noticed. It is safe to say that not one of them has contributed a useful suggestion in behalf of true science, and the appeals for popular effect based upon them, serve far more to bring discredit upon the speciality than for any good purpose. Most of these were observations begun in the ill-informed zeal which attaches to all new methods, and it appears to us without foundation in rational knowledge. Be this as it may, they have been long tried, are entirely without promise of fruit, and may justly be abandoned or greatly modified for this cause.

"In all but the first class, or that of true statistics, according to the division made at the beginning of this article, we have been dealing with proposed aids to the etiology of insanity, although the table of causes is usually only one of twenty special tables in our reports. It will not be necessary to dwell here upon the pre-eminent importance in a medical point of view, of the study of the causes of mental disorder. We are forced to recognise that the morbid phenomena of mind, more than any other class of symptoms which we are called to notice, represent a most profound and radical type of disease. The most marked diatheses, as the scrofulous and nervous, representing the accumulated results of morbid causes perhaps through many generations, may still undergo one further transformation in the downward series,—into mental disease. There are, of course, numerous exceptions, in the temporary disturbance of the cerebral functions by toxic agents, or severe moral shocks, but, as compared with the number of cases in which insanity marks the farthest reach of vital degeneracy, they are but few. To the medical man, therefore, the chief problems of mental disease are of necessity those which refer to its prevention rather than to its cure; and hence the study of causes is of the first and almost exclusive importance.

"But it must be admitted that the manner in which this class of investigations has been pursued, as appears in a large number of asylum-reports, and even of special treatises upon the subject, is the most unpromising, and even absurd, of any arising in the speciality. To say that the certainty with which one phenomenon brings another to succeed it is that which establishes causal relations between them, and not the intimacy of their relation in time or space, seems trite enough; but when we glance at the multitude of hypothetical causes which are stated in our etiological tables, it is sometimes difficult to say what we have better than was found in the days of the belief in astrology and demoniacal possession. If we are to avoid all theory in the observation of causes, and simply to record facts, must we not record all the facts, or how shall we discriminate? In the case of a patient who first exhibits what, in the judgment of the observer, is an insane manifestation during a thunder-storm, is the cause to be given as lightning, or thunder, or tempest, or flood? or is it a moral cause, as anxiety or alarm? Accidents of this sort are constantly stated as causes, and such an instance as the above is by no means an extravagant one. If any of these circumstances are given, certainly none of them should be withheld. But why should the record of any or all of them be the principal, or, as is sometimes the case, the sole, aim of the observer? In a particular case of insanity, we seek the expression of a cause for the sake, mainly, of prognosis; in general, we study etiology in order to find preventive means. It is needless to say that no one seriously supposes meteorological phenomena to contain the elements of a prevision of cerebral symptoms, any more than he does that planetary aspects control the virtues of medicinal herbs. Neither does he intend to urge the necessity of abating thunder-storms, or any of their incidents. We hope to be excused for seeming to trifle here, but we are at a loss how else to comment upon the grave burlesques of scientific forms which the subject calls before us.

"Yet, unfruitful as the etiology of mental disease has hitherto been, and absurd as the tabular forms which have been applied to it, we believe that in the study of development—aided if necessary by tables whose figures may serve to condense simple facts bearing upon some sober hypothesis—is the main hope of progress in mental medicine. It would not probably be necessary, if it were in place, to defend such a belief here, as it has been impressed upon our readers in all the most recent and important works upon insanity. Nor shall we endeavour to give even the outline of a scheme of our own for the study of this all-important but most difficult subject. Certain it is, however, that no single line of inquiry is to be relied upon in so complex and intricate a matter. Our efforts must be moderate and patient. Accepting only the most positive facts, these must be grouped in every possible way, to present the many-sided aspects of cerebro-mental disease. An important step in this direction has lately been made by the celebrated French psychologist M. Morel, in his *Traité des Maladies Mentales*. This author entirely rejects symptoms as the basis of classification in mental disease, and designates the forms of insanity according to causes. Acknowledging the present imperfection of this scheme, he yet, and we think correctly, believes that by thus concentrating the attention of the physician upon the etiology of insanity, a most important point is gained. A brief analysis of his work, in the number of this journal for October, 1860, gives a general idea of the proposed classification, and we hope that the views of M. Morel will gain the attention of every one interested in the progress of psychology. It will be seen at once, how much more condensed, simple, and definite the matter of our tables of causes would become were this method of record adopted. The group 'hereditary insanity' would take the first place, and, through its several classes, receive that especial attention which its great importance is acknowledged to demand. Next in order is 'insanity the effect of toxic agents,' in the widest sense. That all the numerous varieties of this large group must and do tend to develop a certain type of symptoms, will be admitted by the scientific observer. It would comprise narcotic, alcoholic, malarial, puerperal, mineral, and food poisons. The third group, 'insanity caused by the transformation of certain nervous disorders,' would include a large portion of those cases now attributed in such an indefinite way to 'ill health,' and numerous other more or less remote sources. By thus bringing together in large groups the causes of insanity, it will be seen that the tendency is to direct attention mainly to predisposing or efficient causes, to the neglect of the infinite variety of accidental ones.

"But we will not transcend our province by doing more than to point out the direction in which it seems possible some changes in our theory and forms of asylum-statistics might be advantageously made. If it be said that our hints are chiefly in limitation of the present methods, we remember that Drs. Bell and Ray, than whom there are no higher authorities in this or any country upon the subject, have wholly denied the value of the numerical method in insanity, and present only the general statistics of the institutions which have been under their charge. In fact, it is too often found in asylum reports that their professional value is inversely as the length and variety of the statistical tables presented. We do not, however, desire to see all numerical forms abandoned in the study of insanity. The 'medicine of the future,' in many of its departments likely to become almost wholly preventive, and thus of a public and general character, must owe more and more of its progress to statistical science Let us by all means continue such records as have at present any rational value, and let us adopt such other forms as the strict rules of scientific observation will warrant. We may remember that in whatever department rude knowledge has been already developed into science, figures have been without exception the agents of this change. It was by means of a numerical scheme—the atomic theory—that chemistry was raised at once to the rank of an exact science, and the most important applications to medicine and the arts became possible. Indeed, that exactness of any form of knowledge which gives to it the dignity of science exists just in the degree that numerical expression may be given to it. We have no right to deny the possibility that the beneficent aims of the physician may one day be firmly based upon scientific principles, nor to abate the most earnest efforts towards the accomplishment of so important a work."

---

### 3.—*On a Case of Epileptiform Convulsion.* By Dr. Daniel Ayres.

At a meeting of the Brooklyn (U.S.) Medico-Chirurgical Society, in June last, Dr. Ayres brought before the members the subject of the following highly interesting case:—

"In the early part of August last, this lad received a blow from a stone, which inflicted a lacerated wound over the right frontal eminence. His immediate symptoms were those of concussion, followed by vomiting and headache, from which he soon recovered, whilst the wound, after suppurating, healed by granulation, leaving a marked cicatrix, elevated by the permanent thickening and induration of subjacent tissues. In the month of October following, and shortly after the wound had closed, the attention of his family was attracted by the marked change which had taken place in the boy's disposition and character.

"Hitherto he had been peculiarly mild, gentle, and tractable, but gradually became irritable, peevish, morose, and occasionally showed great violence of temper upon the slightest provocation, and a disposition to cry frequently. These paroxysms of excitement were soon accompanied by vertigo and headache, with increased disturbance of his general health. Whilst standing, at such time, he would reel and stagger, occasionally losing all consciousness, and falling down, his countenance becoming very pallid, but no spasm or frothing at the mouth were noticed. During the intervals, his demeanour and aspect were mopish and sluggish; his appetite capricious, and bowels persistingly torpid; his gait was unsteady, with a tendency to trip and drag, rather than raise the left inferior extremity.

"The boy had been very properly removed from school, and submitted to judicious and appropriate medical treatment by his attending physician, Dr. Cullen; but no control was apparently gained over the symptoms, which became steadily more aggravated up to the ensuing February, when he was admitted to the Institute.

"A careful inquiry into his condition and history at that time, seemed to justify the suspicion that the original injury inflicted upon the skull, if not accompanied with fracture of the internal table, had, at least, given rise to serious consecutive changes within, and this suspicion was strengthened by the permanent and decided elevation over the seat of injury. Although unable to define the precise character of this lesion, all were of the opinion that the increasing severity of the symptoms justified an attempt to relieve them by what appeared to be the only rational procedure, viz., the application of the trephine. Perhaps we were more than usually urged to this conclusion by the unfortunate termination of a case of recent occurrence in this city, almost identical in its causes and manifestations, in which the post-mortem examination (kindly furnished by Dr. John Cooper, who witnessed it) revealed a depressed stellated fracture of the internal table, which had gradually superinduced disease of the membranes, and fatal pressure on the brain.

"An operation was accordingly performed on the 9th of February last, by dissecting up a quadrangular flap of integuments, including the cicatrix in its centre. The conical trephine (recently brought again to the notice of the profession by Dr. Galt) was applied, and most satisfactorily fulfilled all the advantages claimed for it. For notwithstanding the youth of the subject, and the unequal thickness of the button of bone (from one-sixteenth to one-eighth of an inch thick), it was quickly removed, in the crown of the trephine, without injury to the dura-mater from the teeth of the instrument. Whatever, therefore, may be urged on the score of originality, Dr. Ayres was confident that no surgeon could employ this valuable contrivance without duly appreciating the sagacity of Dr. Galt.

"After the operation the flap was secured by a suture at each of its inferior angles, thus forming a valvular cover over the opening in the skull, at once excluding external agents, giving vent to exudations, and favouring rapid union. Cold water dressing alone was applied, and the case progressed satisfactorily until the third day, when a violent convulsion attacked the left side of the body, accompanied with smart fever, headache, vomiting, and delirium. Mild purgation, with steady cold to the head, mitigated these symptoms; but the convulsion was repeated on the next day, and then extended to the whole muscular system. Croton oil with sub. myr. hyd., were now administered, until copious alvine results were obtained, and all untoward symptoms speedily disappeared. It was discovered, on inquiry, that he had been allowed full diet through mistake of the nurse, and this important point was sedulously watched and restricted during the subsequent treatment.

"At the end of the third week the wound was entirely closed, and no recurrence of the epileptic symptoms has taken place up to the present time, whilst the patient has entirely regained his former gentle disposition, and, with a good appetite, has wonderfully improved in flesh, strength, and general appearance.

"Dr. Ayres remarked, that whilst we had before us an undoubted case of recovery from a disease, too often beyond control, following an operation justly considered too serious and uncertain in its results to merit trial, except as a dernier ressort, it had stimulated reflection, and given rise to certain conclusions, which, to his mind, constituted the chief features of interest about the case. To recur to the condition of the button of bone removed, it had been found in all respects perfectly normal. The parts beneath were equally without indications of previous injury, or existing disease. How, then, are we to reconcile this total absence of the suspected, or indeed of *any* cause adequate to the production of such morbid phenomena, with the entire success of an operation which revealed nothing, and which, failing in its proposed object, was apparently useless, if not inapplicable?

"This result might be classed in the chapter of accidents, if we had not already too many cases, sufficiently parallel to justify us in seeking for a rational sequence between the operation and the cause of disease.

"Again, the removal of pressure from the brain, by favouring its power of expansion, or, perhaps, allowing some modification of its circulation, has been adduced to account for this dissipation of the epileptic symptoms; but it should not be forgotten that these advantages are seriously curtailed by the tense and unyielding dura-mater when left uninjured. Besides, it will be recollected that this patient suffered his worst epileptic seizures after the bone had been removed. No rational explanation of this paradox occurred to my mind until recently, and I am indebted for it to the experiments of Brown-Séquard. The importance placed by that distinguished physiologist upon what is known as 'the epileptic aura,' and the paramount necessity of patiently and repeatedly searching until the spot is found whence it starts, or seems to take its origin, seemed very applicable to this case; and, from its traumatic origin, very easy, when we came to direct the attention of the patient upon his feelings, for he distinctly recollected that he always felt pain and unpleasant sensations in the vicinity of the cicatrix just preceding the vertigo.

"Then, again, Brown-Séquard has obtained remarkable results from cauterizing this sensitive spot, whereby we may infer that the morbidly sensitive nervous filaments are either destroyed and cut off from their cerebro-spinal connexions, or, at least, so modified in their function that the chain of morbid phenomena is temporarily and, occasionally, permanently broken.

"Is it not, therefore, probable that, to the free dissection of the flap, including the periosteum and cicatrix, we are indebted for the REAL cause of relief in *this* and similar cases? Is not this probability augmented, when we find paroxysms supervening, more severe than on any previous occasion, just at that time when the wound was swollen and about to suppurate; when, in fact, local irritation was at its maximum, and the irritation of undigested food in the alimentary canal was superadded?

"We think the sequence is complete, and the deduction logical, that in all such cases, before hazarding the serious operation of trephining, the superficial tissues which have been the subject of injury should be freely separated from the parts beneath and around, by subcutaneous division. This plan failing, a portion, sufficient to include all the injured nerves, should be freely dissected out, and its place supplied by sliding together the contiguous tissues, or by a patch transposed from neighbouring parts.

"In the discussion which followed, Dr. Lewis A. Sayre stated that nearly a year had elapsed since he performed a similar operation upon an adult, on account of epilepsy following a blow received many years previously on the occipital region, and immediately over the longitudinal sinus. The case had resisted all medical treatment, and he was a confirmed epileptic up to the time of operation. Although the disease had not since returned, yet he had been very much disappointed at the time, and had ever since been at a loss to account for the relief thus obtained, inasmuch as the portion of skull removed by the trephine was found absolutely free from exostosis or any other morbid change. He considered the explanations offered by Dr. Ayres as highly probable, and calculated to render our treatment of these cases equally successful, without danger to the patient.

"Dr. Andrew Otterson remarked that the relation of these cases brought to his recollection the case of a sailor who suffered from epilepsy following an injury, upon whom Dr. Buck operated several years ago. When the bone was removed no disease was found, yet the patient recovered, and he learned on inquiry a long time afterwards, that no return of the epileptic attacks had taken place."

